# Dietary Regulation of Memory T Cells

**DOI:** 10.3390/ijms21124363

**Published:** 2020-06-19

**Authors:** Nicholas Collins

**Affiliations:** Metaorganism Immunity Section, Laboratory of Immune System Biology, National Institute of Allergy and Infectious Diseases, National Institutes of Health, Bethesda, MD 20892, USA; Nicholas.collins@nih.gov

**Keywords:** immune system, memory T cell, vaccination, cancer immunotherapy, diet, nutrition, caloric restriction, undernutrition, diet-induced obesity, fiber, microbiota

## Abstract

Memory T cells are a fundamental component of immunological memory, providing rapid and potent host protection against secondary challenges. As such, memory T cells are key targets in the design of vaccination strategies and cancer immunotherapies, making it critical to understand the factors and mechanisms that regulate their biology. Diet is an environmental feature that impacts virtually all aspects of host physiology. However, the influence of specific dietary regiments and nutritional components on the immune system is only just starting to be uncovered. This article will review literature regarding the impact of diet and nutrition on memory T cell development, maintenance and function. It was recently shown that caloric restriction without undernutrition enhances memory T cell function, while diets high in fiber are also beneficial. However, memory T cell responses are dysfunctional in extreme nutritional states, such as undernutrition and diet-induced obesity. Therefore, diet and host nutritional status are major regulators of memory T cell biology and host fitness. To define the dietary balance required to promote optimal memory T cell responses could allow for the implementation of rational diet-based therapies that prevent or treat disease. Furthermore, that certain dietary regiments can enhance memory T cell function indicates the possibility of harnessing the underlying mechanisms in the design of novel vaccination strategies and cancer immunotherapies.

## 1. Introduction

During an immune response, antigen-specific naïve T cells interact with antigen-presenting cells displaying cognate antigen in the context of major histocompatibility molecules, resulting in extensive T cell proliferation and the acquisition of an effector program [1]. Following control of the threat, the majority of effector T cells undergo apoptosis, leaving behind stable populations of memory T cells with the potential to confer life-long immunity [1]. Memory T cells have traditionally been partitioned into subsets based on migratory capacity and type of effector functions exhibited upon secondary challenge [1,2]. These subsets include the continuously patrolling circulating populations that provide body-wide immunosurveillance, comprised of central (T_CM_) and effector (T_EM_) memory T cells [3]. T_CM_ circulate between secondary lymphoid organs (SLO), blood and lymphatics. Upon activation these cells are highly proliferative, contributing secondary effector cells to combat the challenge. Conversely, T_EM_ circulate between peripheral tissues and blood, with low proliferative capacity but the ability to provide immediate effector functions. These functions include the production of pro-inflammatory cytokines and direct killing of target cells via the expression of cytotoxic granules. In contrast, tissue-resident memory T cells (T_RM_) are non-migratory, persisting long-term in tissues without re-entering the circulation [2]. These cells rapidly produce cytokines that initiate and amplify local immune responses, thereby providing exposed sites with an essential layer of protection [2,4,5,6]. In addition, other subsets such as stem cell (T_SCM_) [7], recirculating (T_RCM_) [8] and peripheral (T_PM_) [9] memory T cells have been described, with unique features that distinguish them from the already mentioned subsets (Table 1). Considerable plasticity between subsets has also been demonstrated, with resident and circulating cells having the potential to interchange [10]. Thus, the memory T cell compartment consists of a dynamic blend of complementary cells that protect the host against a broad array of local and systemic challenges [4].

Memory T cell development from activated effector cells is thought to be regulated by several elements, including the strength of signaling received via the T cell receptor during activation, as well as exposure to inflammatory factors over the course of the response [1]. The long-term maintenance of memory T cells does not require antigen but is dependent on specific transcription factors and the homeostatic cytokines IL-7 and IL-15, which promote a quiescent, pro-survival program [11,12,13,14]. IL-7 signaling is critical for many aspects of memory T cell biology, such as promoting the processes by which metabolic fuels are oxidized within the mitochondria to generate energy in the form of ATP [15]. The work of several groups has demonstrated that oxidative phosphorylation of long-chain fatty acids (LCFA) is likely to represent the preferential, although redundant, program employed to support memory T cell development, maintenance and function [15,16,17,18,19,20,21]. Collectively, the abovementioned studies have uncovered many critical features regarding resting memory T cells within hosts at steady state. However, the host is likely to be exposed to a fluctuating environment over time, with alterations in caloric intake and dietary composition a common occurrence throughout evolutionary history. How such events impact the immune system and particularly the memory T cell compartment are just beginning to be characterized [22,23,24].

## 2. The Impact of Caloric Restriction on Memory T Cells

Organisms likely experienced prolonged periods of reduced food availability throughout history. As such, having the ability to adapt and thrive when food became scarce may have represented a major aspect in shaping human evolution. Caloric restriction (CR) refers to reduced caloric intake without undernutrition or deficiency in vitamins, minerals or amino acids [22,25]. It has been appreciated for decades that CR promotes host fitness in organisms spanning from yeast to humans [25]. Benefits include extending longevity, improving metabolic profiles, as well as reducing cardiovascular disease, neurodegeneration, basal levels of inflammation, and the incidence of certain types of cancer [25,26,27,28,29,30,31,32]. It was recently shown that CR also has a dramatic impact on the migration, intrinsic cellular state and functional capacity of established CD4^+^ and CD8^+^ memory T cells [24]. As little as one week of 50% CR in mice induced the redistribution of circulating memory T cells from secondary lymphoid organs and blood, sites that are usually dense with T cells, to the bone marrow (BM). Of note, naïve B cells [33] and monocytes [34] were also recently shown to accumulate in BM during fasting, indicating that this niche may act as a “safe haven” [23] or “metabolic refuge” [35] for cells of the immune system when caloric intake is reduced. Memory T cell redistribution was coordinated by the steroid hormones glucocorticoids, which are known to induce expression of the BM-homing receptor CXCR4 on T cells [36,37,38,39]. In parallel, the BM was drastically remodeled during CR to be enriched for T cell trophic factors, red blood cells and adipocytes, all of which worked in concert to recruit, retain and protect memory T cells [24]. BM adipocytes are a critical source of hormones during CR that compensate for the loss of peripheral white adipose tissue (WAT) in this context [40,41,42,43]. Peripheral WAT is a major site of memory T cell lodgment following the clearance of an infection [44,45,46,47]. When in peripheral WAT, memory T cells show increased homeostatic proliferation, enhanced effector potential and utilization of fatty acids, indicating that unique interactions occur within this site that support a heightened level of memory T cell homeostasis [44]. Memory T cells could not accumulate in BM efficiently if adipocytes were ablated during CR [24], suggesting that BM adipocytes support memory T cell survival or persistence within this niche. However, memory T cells from mice on CR showed a similar ability to uptake, store and utilize fatty acids compared to memory T cells from mice fed *ad libitum* [24]. Therefore, the mechanisms by which BM adipocytes support memory T cells remains an open question and is an ongoing area of research [24]. While memory T cells did not show signs of altered fatty-acid metabolism during CR, these cells were in a particularly quiescent state. Their cellular profile was associated with reductions in motility, homeostatic proliferation, mitochondrial activity and signaling via the mechanistic target of rapamycin (mTOR) during CR [24]. mTOR is an evolutionary conserved nutrient sensor that stimulates cell growth when nutrients are abundant and promotes quiescence when nutrients are limited [48]. Although in a state of reduced metabolic activity during CR, memory T cell function was markedly enhanced [24]. This resulted in superior protection against secondary bacterial infections and tumors, greatly prolonging host survival [24]. Such findings are consistent with a separate study showing that CR enhanced influenza-specific memory T cells in terms of their proliferative capacity and ability to produce effector cytokines [49]. Although CR induces a number of beneficial changes to host physiology, several studies suggest that reduced mTOR signaling could be central to enhancing memory T cell function in this context. Low-dose treatment with rapamycin, which pharmacologically reduces mTOR signaling and induces cells into a state of CR, is sufficient to enhance memory T cell development, maintenance and protective function in the context of viral infection [50]. Furthermore, melanoma-specific CD8^+^ T cells cultured in vitro under conditions that induce ‘functional caloric restriction’ showed reduced mTOR signaling and mediated striking tumor control following adoptive transfer into mice [51]. In addition, compounds that reduce mTOR signaling have shown promise in the clinic in the context of vaccine responses [52]. Together, several lines of research support the notion that CR promotes memory T cell function to mediate host protection against secondary challenges, which may be regulated by the mTOR pathway.

Overall, these studies highlight the ability of memory T cells to rewire in response to reduced calorie availability to not only persist, but to thrive. This raises questions regarding the optimal host state for promoting highly functional immune responses. Relatively low levels of food availability compared to the standards of today was likely the case for the vast majority of human evolution. Therefore, it may be that low food intake, with sufficient nutrition, is the ideal state for promoting not only longevity and general health profiles [25,27,29], but also optimal memory T cell function. However, much remains to be uncovered if CR itself, or the mechanisms by which CR enhances T cell function, are to be harnessed therapeutically in the design of novel vaccination strategies and cancer immunotherapies. For example, the metabolic pathways engaged and fuel sources utilized by memory T cells during CR remain unclear, with recent advances in characterizing T cell metabolism in vivo likely to aid in addressing this open question [53]. The minimum duration and degree of CR required to promote beneficial effects on the memory T cell compartment has not been systematically evaluated, nor has it been determined whether less acute forms of dietary intervention such as intermittent fasting [54] are similarly beneficial. It will also be of importance to determine the optimal age at which to initiate CR, as it has been shown that benefits on the T cell compartment only manifest if CR is implemented when the host is relatively young, with T cells from elderly hosts unaffected by CR [55].

## 3. The Impact of Undernutrition and Reductions in Dietary Metabolites on Memory T Cells

While reduced caloric intake with adequate nutrition promotes memory T cell function, undernutrition will ensue if food intake is severely reduced, with highly detrimental consequences for immunity [56]. Undernutrition affects more than 800 million people globally and is associated with increased susceptibility to infection, as well as a reduction in the titer and persistence of antibody responses elicited to certain vaccines [56,57,58]. Accordingly, undernutrition has been described as the dominant cause of immunosuppression worldwide [58]. It is a complex condition, with poor nutrition regularly associated with chronic infections, a state of low-grade inflammation, increased intestinal permeability and dysbiosis of the intestinal microbiota [56,59,60]. Therefore, there are difficulties in assessing the contribution of each individual aspect to weakened immune responses in undernourished individuals. Nonetheless, it has been shown that dietary protein plays a major role in regulating memory T cell development, maintenance and function. A severe reduction in dietary protein (0.6% protein compared to 18% in the control diet) resulting in protein energy malnutrition (PEM) was shown to drastically reduce the ability of established memory CD8^+^ T cells to persist long-term [61]. This was due to a reduction in proliferative potential, both homeostatically and in response to an inflammatory stimulus. Consequently, mice in a state of PEM were more susceptible to secondary infections in a model of lymphocytic choriomeningitis virus (LCMV) [61]. A separate study also found suppressed immune responses when investigating the impact of PEM on vaccine-elicited *Mycobacterium tuberculosis (M-tb)*-specific memory CD4^+^ T cells [62]. *M-tb* was controlled long term in the lungs of vaccinated mice fed a regular diet. However, mice fed a regular diet during the vaccination phase, then transferred to diet that induced PEM following the establishment of memory, showed an inability to control the bacterium and exhibited severe lung pathology [62]. Importantly, memory CD4^+^ T cell in the lungs of mice suffering from PEM were compromised in their ability to produce effector cytokines, indicating the critical role of dietary protein in supporting the function of memory T cells [62]. These studies demonstrated the impact of PEM on hosts that were initially infected or vaccinated when fed a regular diet. Memory T cell development was therefore not influenced in these cases. However, two separate investigations showed that mice fed diets very low in protein prior to and during a primary infection were impaired in their ability to generate effector T cells in models of influenza virus and LCMV [63,64]. Therefore, as memory populations are thought to develop via an effector T cell stage [65,66], it is possible that diets inducing PEM negatively impact both memory T cell maintenance and development. Future studies exploring the dynamic between PEM and other common features of undernutrition, such as an altered intestinal microbiota and increased gut permeability [60], on memory T cell biology will be of interest. Furthermore, establishing whether reductions in certain amino acids are sufficient to drive the memory T cell phenotypes observed in PEM could result in the development of novel therapies that restore suppressed immunity in the setting of undernutrition.

The critical role of specific metabolites in T cell activation and memory T cell development has become appreciated in recent years. Reductions in dietary metabolites may therefore be the cause of, or at least contribute to, defective immunity in undernourished individuals. The amino acid serine was shown to be required for effector CD8^+^ T cell proliferation and function [53,67]. T cell activation in the context of a serine-free diet resulted in reduced memory development, leading to inefficient pathogen control upon secondary challenge [67]. Mechanistically, serine was required for nucleotide synthesis during proliferation, highlighting the fundamental role of this metabolite in T cell biology [67]. Other individual metabolites that promote T cell activation include methionine [68]. Mice fed a diet with reduced levels of this amino acid showed restricted CD4^+^ T cell activation and differentiation, due to altered regulation of chromatin accessibility and gene expression [68]. Furthermore, the amino acid L-Arginine was shown to be essential in regulating several metabolic pathways in CD4^+^ T cells [69]. High levels of intracellular L-arginine increased mitochondrial metabolism, which promoted T cell survival and differentiation into T_CM_ that had potent anti-tumor activity [69]. L-arginine could be administered as a dietary supplement to mice fed a regular diet to promote differentiation into this beneficial state [69]. As such, it could be that a lack of dietary L-arginine results in suboptimal immune responses, although this remains to be formally investigated.

Collectively, while memory T cells can adapt and thrive in the context of CR when nutrition is sufficient, the survival and function of these cells is compromised when specific dietary components are very low or absent. Such results could provide direction with regard to the design of dietary supplement regimes aimed at restoring immunity in undernourished individuals. Furthermore, if CR is to be utilized therapeutically, this information must be incorporated so that sufficient buffers are in place to avoid the extremely negative consequences of undernutrition-induced immunosuppression.

## 4. The Impact of Diet-Induced Obesity on Memory T Cells

At the opposite end of the scale of undernutrition is obesity. The ability to constantly access and excessively consume food with a high fat content has likely arisen very recently in human history [70]. Obesity is associated with metabolic syndrome and chronic low-grade inflammation, both of which are detrimental to several aspects of host physiology [71]. For example, diet-induced obesity increases the risk of developing cardiovascular disease, certain types of cancer and type two diabetes [71,72]. In terms of infectious disease, obesity was shown to be an independent risk factor in promoting morbidity and mortality following infection with the H1N1 2009 pandemic strain of influenza virus [73,74,75]. In addition, obesity is associated with suboptimal antibody responses and suppressed memory T cell function following vaccination [72,76,77,78,79,80]. However, despite these observations suggesting deleterious consequences, the impact of diet-induced obesity on the ability of memory T cells to respond to secondary infections is not entirely clear at present.

One study showed that mice fed a diet in which approximately 60% of calories were derived from fat (sources of fat: 93% coconut oil and 7% soybean oil) exhibited obesity, which increased mortality upon secondary infection with influenza virus [81]. This correlated with a subtle reduction in number of functional CD8^+^ T_EM_ in the lungs [82]. T_CM_ were not affected [81], suggesting a potential defect in the maintenance of certain memory T cell subsets in this context. Of potential interest, mice fed a high fat diet, then re-fed a control diet went on to lose a significant amount of weight but did not return to baseline, which was associated with persistent alterations in memory T cell function and metabolic state [83]. Such findings suggest that a period of obesity could have a long-term impact on host physiology and the immune system even after switching to a healthier diet. Having said that, whether diet-induced obesity negatively impacts the ability of memory T cells to control secondary infections in mice has not been consistent between studies. For example, another study where mice were also fed a diet in which 60% of calories were derived from fat (sources of fat: 91% lard and 9% soybean oil) did not observe any impact on memory T cell development, maintenance or function in response to vaccination, influenza virus or a bacterial pathogen [84]. Furthermore, obese mice primed and challenged with strains of influenza virus different from that in the studies described above actually showed increased numbers of memory T cells in the lungs during a secondary response [85]. In this latter study, mice were obese, although the high fat diet used consisted of 45% of calories being derived from fat (sources of fat: 88% lard and 12% soybean oil) instead of 60%. As noted, there is a lack of standardization in the high fat diets used in these studies. While the high fat diets all induced obesity, they consisted of different sources of fat or different ratios of fats to carbohydrates. This raises the possibility that distinct sources of fat can differentially regulate memory T cell biology. As such, future studies that directly compare the impact of diets with different fat contents, as well as distinct fat sources, on the memory T cell compartment could help in clarifying the discrepancies discussed above. Thus, while diet-induced obesity clearly predisposes individuals to an increased risk of morbidity and mortality during influenza infection, and suppresses responses following vaccination, its specific impact on memory T cells in these contexts remains to be uncovered.

Separately to pathogens that cause lung infections, a detrimental impact of diet-induced obesity on memory T cells has been described in the context of pathogens that directly infect adipocytes. It was recently shown that LCMV infects WAT and was cleared by effector T cells in mice fed either a regular or high fat diet (60% fat, of which 91% of the fat was derived from lard and 9% from soybean oil) [45]. Memory T cells were then generated and persisted long-term in WAT, consistent with previous reports [44]. Strikingly, WAT memory T cells of obese mice caused lethal immunopathology upon a secondary infection, whereas mice on a regular diet were unaffected and could efficiently control the challenge [45]. Mechanistically, memory T cell-dependent destruction of infected adipocytes in obese mice resulted in necrosis of WAT and the release of lipases and other factors, causing inflammation in regions of the pancreas adjacent to WAT [45]. These results suggested that diet-induced obesity caused an aberrant inflammatory program within WAT memory T cells. In support of this, innate immune cells in WAT of obese mice show increased production of pro-inflammatory factors that contribute to dysregulated host responses [71]. Thus, while memory T cells in adipose tissue of lean mice play a role in host protection and in aligning the profile of adipocytes to promote pathogen control [44], this is clearly dysregulated during obesity, with lethal consequences in the context of pathogens that directly infect adipocytes [45]. An altered, pro-inflammatory profile has also been observed in memory CD4^+^ T cells that develop in obese mice (sources of fat not described in the high fat diet used) [86]. This profile was directly induced by the long-chain fatty acid palmitate, which led to increased activation of a PI3K p110d-AKT-dependent pathway and supported memory T cell migration to inflamed non-lymphoid tissues [86]. Although the response to secondary infections was not examined in this study, the altered memory T cell program promoted the rejection of skin grafts [86]. Dysfunctional T cell responses during diet-induced obesity have also been found in the context of cancer [87]. Intra-tumoral T cells were highly susceptible to becoming exhausted and less functional in obese hosts (fed a diet with 60% of calories derived from fat, of which 91% was from lard and 9% from soybean oil), which was associated with accelerated tumor growth [87]. Unexpectedly, obese mice were more responsive to treatment with immunotherapeutic agents [87], such as anti-programmed cell death protein 1 (PD1) therapy, which reinvigorates the function of exhausted T cells [88]. Increased responsiveness to anti-PD1 therapy enhanced tumor control in obese hosts compared to mice fed a regular diet [87]. This was also shown to be the case for obese humans [87], suggesting a potential beneficial by-product derived from the pro-inflammatory profile of T cells in the context of diet-induced obesity.

Overall, the current data suggests a picture of dysfunctional, aberrant T cell activation and memory T cell responses in the context of diet-induced obesity. However, whether this has a consequence for the ability of memory T cells to control secondary infections is unclear. The pro-inflammatory profile of memory T cells in the context of obesity appears to promote immunopathology, while also conferring a beneficial by-product in the setting of cancer immunotherapy. Having constant access to and excessively consuming food with a high fat content is likely to be a very recent phenomenon in evolutionary history. As the physiology of the vast majority of present-day humans did not evolve in such a context [70], it is not surprising that consuming high amounts of these foods has consequences with regard to the development and outcome of disease. However, more work is required to clearly determine the impact of diet-induced obesity on memory T cells in a range of contexts.

## 5. The Impact of Dietary Fiber on Memory T Cells

Dietary intake and composition are major causes of alteration to the intestinal microbiota [89]. The microbiota includes trillions of beneficial bacteria that have co-evolved with humans and impact virtually all aspects of host physiology [90,91]. A key quality of the intestinal microbiota is the ability to digest components of dietary fiber into short chain fatty acids (SCFAs) [92,93]. Consuming a diet high in fiber is associated with many health benefits, such as protection against obesity, colon cancer, duodenal ulcers, diabetes, stroke, hypertension and cardiovascular disease [94]. With regard to the immune response, the SCFAs acetate, propionate and butyrate are produced in abundance by the microbiota and possess potent immunomodulatory effects that are critical for development and function of various populations of CD4^+^ T cells [93]. It has recently become clear that the microbiota is also required to support the development of memory CD8^+^ T cells [95]. This was shown by feeding mice a diet high in fiber (35% crude fiber compared to 5% in the control diet), which resulted in enhanced memory T cell development and functionality in the context of a herpes-simplex virus infection [95]. The microbiota was essential for this process, as effector CD8^+^ T cells could not efficiently develop into circulating memory cells in germ-free mice that lack a microbiota [95]. Furthermore, the action of butyrate was critical, by promoting mitochondrial oxidative metabolism in effector T cells [95,96], which is necessary to induce a memory program [97,98]. In addition, butyrate has been shown to directly enhance effector CD8^+^ T cell function by regulating chromatin accessibility and promoting the expression of genes that encode effector molecules [99].

Together, while the effects of the microbiota in regulating host physiology are broad and numerous, the impact of the microbiota on memory T cells is only starting to be uncovered. Future studies determining the impact of the microbiota on T_RM_ will be of interest. These highly functional cells are found throughout the body [100] but are particularly enriched within epithelial layers of barrier tissues such as the intestine, lung, reproductive tract and skin [5,46,101], sites densely populated with commensal bacteria. Furthermore, the impact of dietary fiber on the memory T cell compartment via the microbiota raises questions with regard to the contribution of the microbiota in regulating memory T cells during other dietary states. This is indeed possible, with drastic changes in the intestinal microbiota shown to occur in contexts of a high fat diet [102,103], undernutrition [56,60] and caloric restriction [104,105]. Therefore, it will be important for future studies to incorporate the interplay between diet and the microbiota in regulating immune responses throughout various nutritional states.

## 6. Conclusions

**Figure 1:**
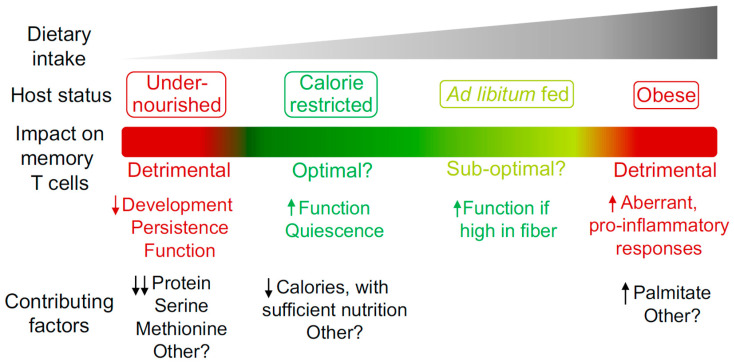
The impact of host nutritional state on the memory T cell compartment. Different degrees of caloric intake can contribute to the induction of specific host nutritional states. These states can have a beneficial or detrimental impact on the memory T cell compartment in terms of development, persistence and function. Some of the known dietary components that contribute to the regulation of memory T cells are listed.

Dietary intake and composition have a major impact on host physiology. However, much remains to be uncovered in terms of the mechanistic processes by which diet regulates memory T cell biology. While memory T cells show an ability to adapt and thrive in the context of CR without undernutrition, this phenomenon has its limits, with detrimental effects observed when specific dietary components are absent, reduced or excessive. Such findings could indicate that reduced caloric intake with sufficient nutrition is the optimal state for promoting memory T cell responses, with this context likely to have been dominant for the vast majority of evolutionary history. Future studies defining the level of caloric intake and balance of nutritional components required to promote optimal memory T cell development, persistence and function (Figure 1) could influence the development of rational diet-based therapeutic options that prevent or treat infectious disease and cancer. In the context of cancer, diet-based treatments could be used in isolation or in combination with other existing therapies such as chemotherapy, radiotherapy or immunotherapy [28,106,107]. However, virtually all studies investigating the impact of diet on the memory T cell compartment have focused on circulating subsets. To design optimal therapies, it will be of importance to determine if similar processes also occur in T_RM_, T_SCM_, T_PM_ and T_RCM_, which are regulated by different factors but must work collectively to ensure optimal host protection [2]. Furthermore, the ability of diet to enhance memory T cell function indicates the potential to harness the underlying mechanisms in the design of novel vaccination strategies and cancer immunotherapies.

## Figures and Tables

**Table 1:** Location and function of memory T cells.

Subset	Location	Function
**T_CM_ [3]**	Circulation, SLO, BM	Proliferation, persistence
**T_EM_ [3]**	Predominantly blood, also peripheral tissues	Cytokine production, cytotoxic activity
**T_RM_ [2]**	Peripheral tissues	Cytokine production
**T_PM_ [9]**	Predominantly peripheral tissues, also SLO and circulation	Cytokine production
**T_RCM_ [8]**	Peripheral tissues, SLO, circulation	Cytokine production (particularly IL-2)
**T_SCM_ [7]**	SLO, circulation	Superior proliferation and persistence
